# Motor Network Degeneration in Amyotrophic Lateral Sclerosis: A Structural and Functional Connectivity Study

**DOI:** 10.1371/journal.pone.0013664

**Published:** 2010-10-27

**Authors:** Esther Verstraete, Martijn P. van den Heuvel, Jan H. Veldink, Niels Blanken, René C. Mandl, Hilleke E. Hulshoff Pol, Leonard H. van den Berg

**Affiliations:** 1 Department of Neurology, Rudolf Magnus Institute of Neuroscience, University Medical Centre Utrecht, Utrecht, The Netherlands; 2 Department of Psychiatry, Rudolf Magnus Institute of Neuroscience, University Medical Centre Utrecht, Utrecht, The Netherlands; 3 Department of Radiology, University Medical Centre Utrecht, Utrecht, The Netherlands; University of California San Francisco, United States of America

## Abstract

**Background:**

Amyotrophic lateral sclerosis (ALS) is a neurodegenerative disease characterised by motor neuron degeneration. How this disease affects the central motor network is largely unknown. Here, we combined for the first time structural and functional imaging measures on the motor network in patients with ALS and healthy controls.

**Methodology/Principal Findings:**

Structural measures included whole brain cortical thickness and diffusion tensor imaging (DTI) of crucial motor tracts. These structural measures were combined with functional connectivity analysis of the motor network based on resting state fMRI. Focal cortical thinning was observed in the primary motor area in patients with ALS compared to controls and was found to correlate with disease progression. DTI revealed reduced FA values in the corpus callosum and in the rostral part of the corticospinal tract. Overall functional organisation of the motor network was unchanged in patients with ALS compared to healthy controls, however the level of functional connectedness was significantly correlated with disease progression rate. Patients with increased connectedness appear to have a more progressive disease course.

**Conclusions/Significance:**

We demonstrate structural motor network deterioration in ALS with preserved functional connectivity measures. The positive correlation between functional connectedness of the motor network and disease progression rate could suggest spread of disease along functional connections of the motor network.

## Introduction

Amyotrophic lateral sclerosis (ALS) is a devastating disease characterised by degeneration of motor neurons in the brain and spinal cord leading to progressive muscle weakness. With a prevalence of approximately 6/100.000 it is considered a rare disease. The pathogenesis of ALS is heterogeneous with monogenetic causes in familial ALS and environmental and genetic risk factors in sporadic ALS [1]–[3]. The natural history shows a large variablity. With a median survival of three years it is intriguing that a minority of patients survive more than ten years. Currently we are unable to predict the disease course in the individual patient. Regardless a few clinical prognostic indicators it is largely unknown which factors determine the rate of clinical decline. We hypothesized that motor network characteristics could influence its vulnarability to neurodegenerative effects.

Previous structural imaging studies on either gray or white matter in patients with ALS have revealed signs of upper motor neuron degeneration in vivo [4]–[12]. A limited number of functional imaging studies have reported altered functional neuronal activity of central motor regions [13]–[16]. These neuroimaging studies have, however, focused on discrete measures and lack the network perspective in combination with clinical markers.

**Figure 1:**
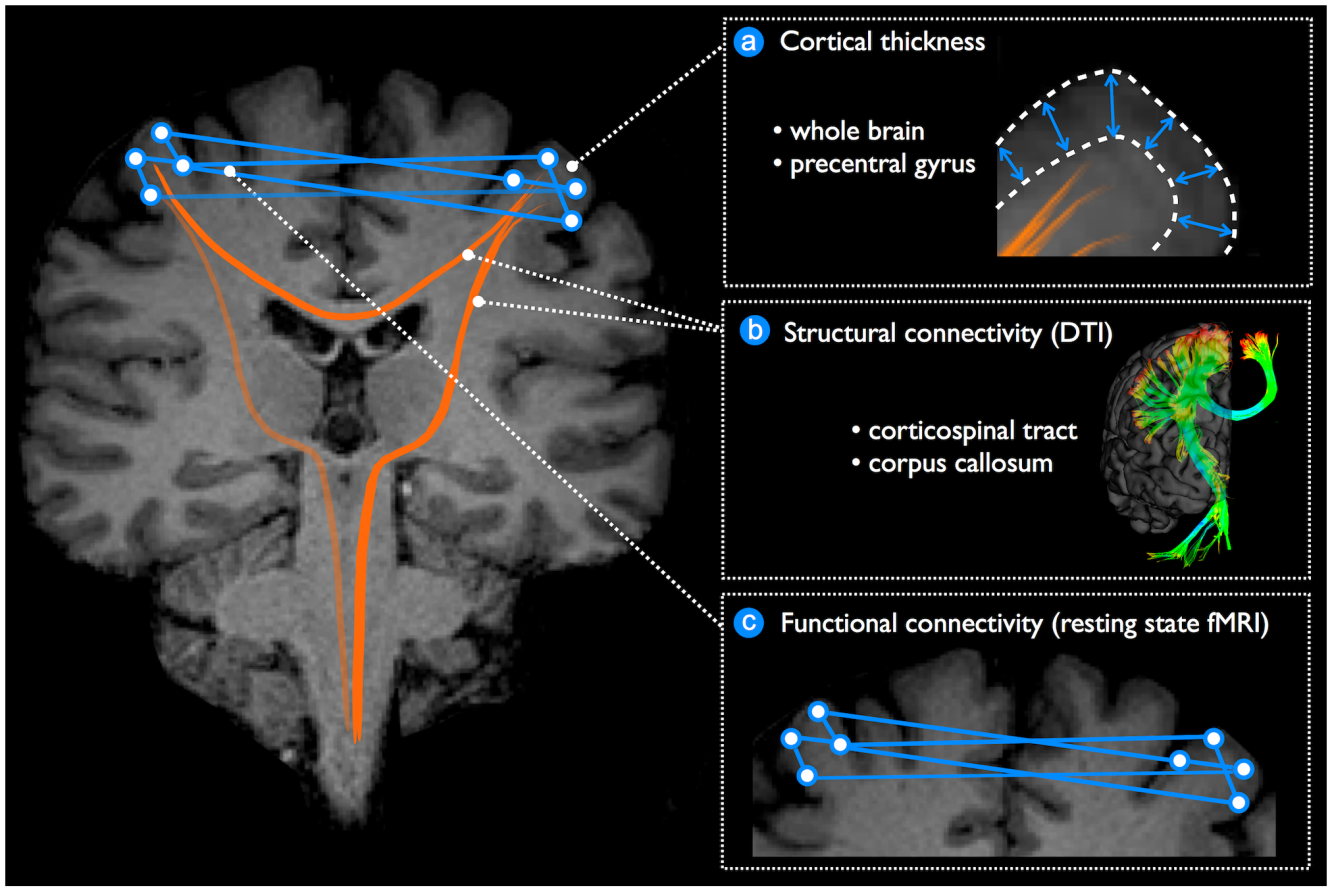
Schematic overview of the performed measures.

A neural network can be defined as a population of interconnected nodes that perform a specific physiological function, just as the motor network controls muscle tone and movement. The properties and integrity of cortical neural networks can be explored using structural or functional measures. We performed cortical thickness measures to assess the quality of the computational units (nodes) in the neural network (Figure 1a) and Diffusion Tensor Imaging (DTI) to explore the integrity of the interconnecting white matter tracts (structural connectivity) (Figure 1b). DTI is a technique that measures the water diffusion profile in brain tissue. This technique enables the reconstruction of white matter tracts and estimates its microstructural integrity, which is typically expressed as the fractional anisotropy (FA). As fibres degenerate, diffusion becomes less directional and the FA decreases [17]. We studied the functional characteristics of the motor network by resting-state functional MRI (rs-fMRI). rs-fMRI is designed to detect inter-regional correlations in spontaneous neuronal activity as measured by blood oxygen level-dependent (BOLD) signal fluctuations over time. This phenomenon was first described in the motor system as a manifestation of functional connectivity [18]. Interestingly, recent studies have shown that functional connections in neuronal networks have a structural core of white matter [19]–[22]. Combining these techniques in neurodegenerative diseases which typically affect a specific neural network, as ALS affects the motor network, has not been reported previously but is promising future direction [23].

We hypothesized cortical atrophy to occur mainly in primary motor areas with conjoint loss of motor tract integrity. The degree of structural motor network degeneration was expected to affect the functional connectivity. Finally, both structural and/or functional motor network decline was hypothesized to correlate with the clinical status. Here, we examined the neurodegenerative effects in patients with ALS by investigating the structural and functional motor network characteristics and we explored their link with clinical markers.

## Results

### Cortical thickness

**Figure 2:**
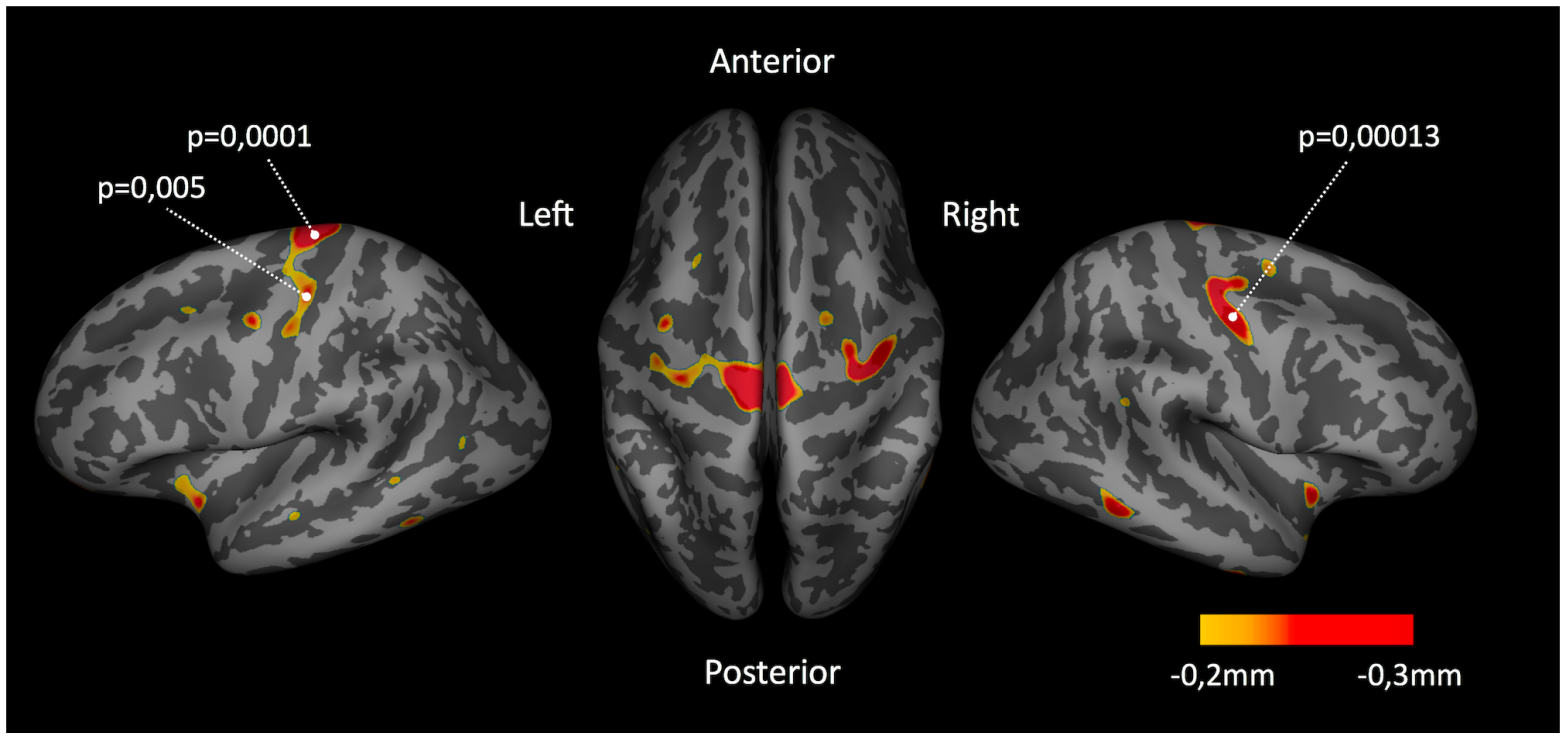
Whole brain cortical thickness. Whole brain cortical thickness measurements in patients with ALS compared to healthy controls. Cortical areas with (uncorrected) p-values <0.01 are marked. The threshold for this illustration was set at 0.2 mm cortical thickness reduction in ALS.

**Figure 3:**
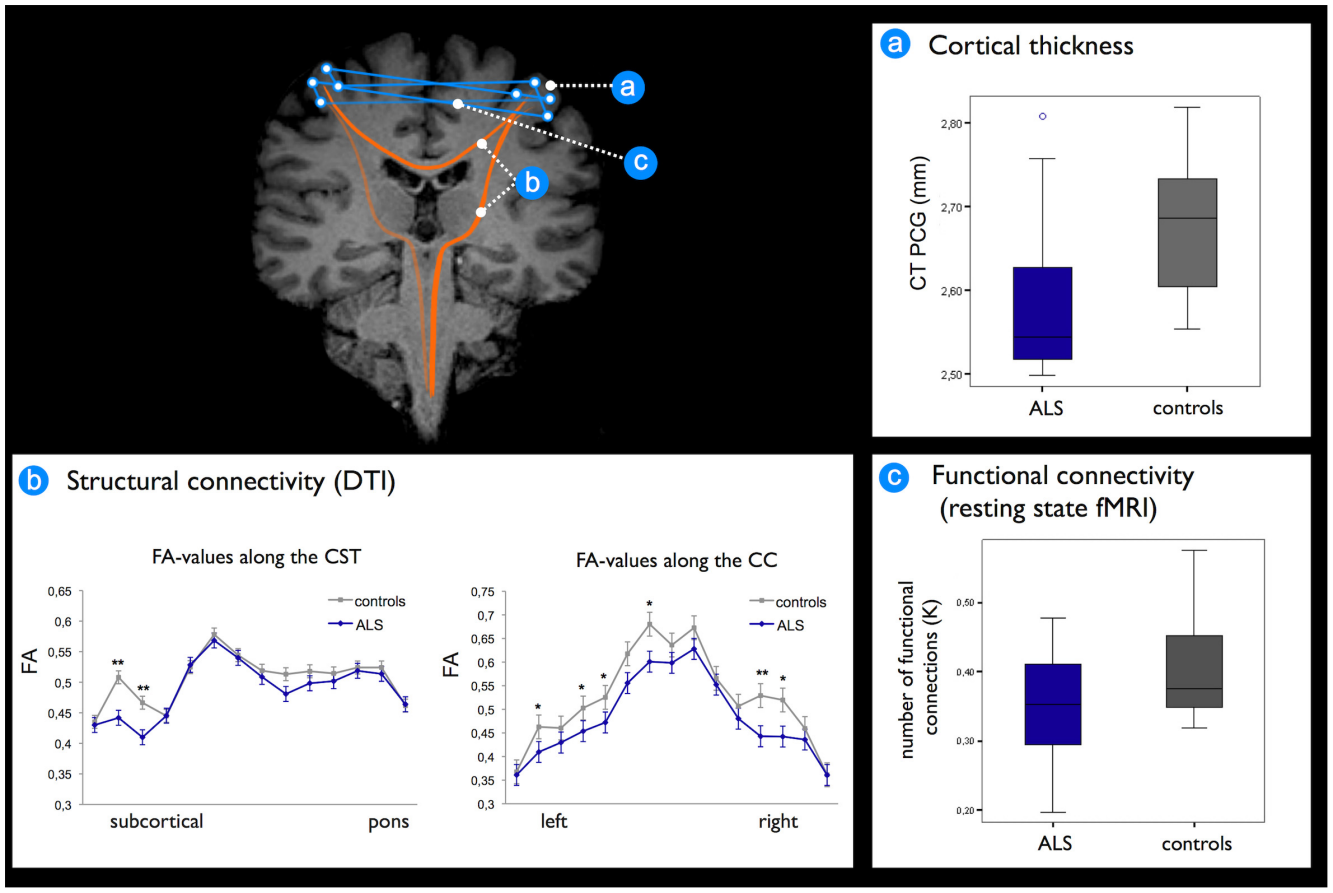
Overview of motor network measures. a) Cortical thickness in patients with ALS versus controls in the precentral gyrus in mm (p = 0.04), corrected for age and whole brain cortical thickness; b) Fractional anisotropy (FA) values along the corticospinal tract and the corpus callosum in patients with ALS and controls (** p<0.01; * p<0.05); c) Number of functional connections, corrected for age (threshold 0.40); p = 0.14, CT = cortical thickness. PCG = precentral gyrus. FA = fractional anisotropy.

Whole vertex-wise brain comparisons revealed cortical thinning in the precentral gyrus in patients with ALS compared to healthy controls as illustrated in Figure 2 (left and right precentral gyrus: p<0.0001 and p<0.001, respectively). Focussing on the precentral gyrus most pronounced thinning in ALS was found bilaterally in the two subareas overlapping the central motor representations of the hand and leg. Interestingly, thinning of the primary motor regions was found to be specific, as no other cortical regions showed any changes in cortical thickness (p<0.01). Indeed, post-hoc analysis of whole brain average cortical thickness revealed that ALS patients showed no differences in whole brain average cortical thickness (mean 2.278 mm; SD 0.090 mm) compared to controls (mean 2.271 mm; SD 0.0449 mm; p = 0.81). Furthermore, focussing on the primary motor network, the average cortical thickness of the precentral gyrus as a more broadly defined anatomical region was significantly reduced in patients with ALS compared to healthy controls (ALS mean 2.589 mm; SD 0.120 versus controls mean 2.680 mm; SD 0.087; p = 0.04) (Figure 3a).

### Structural connectivity

FA values in the corpus callosum and the CST were found to be significantly reduced (p<0.05) in ALS patients compared to controls (Figure 3b). The FA reduction was more dispersed in the corpus callosum compared to the CST. Tract-based results, analysing the trajectory of FA along the CST tracts, showed that the reduction in FA becomes less pronounced as the tract descends from the cortex to the brainstem corresponding to more loss of integrity in the rostral part compared to the caudal part of the CST (Figure 3b). The FA along the visual tracts as a negative control condition was found to be unchanged in patients with ALS (0.583) compared to healthy controls (0.576) (p = 0.07). The whole brain average FA was 0.453 in patients and 0.458 in controls (p = 0.48).

### Functional connectivity

**Figure 4:**
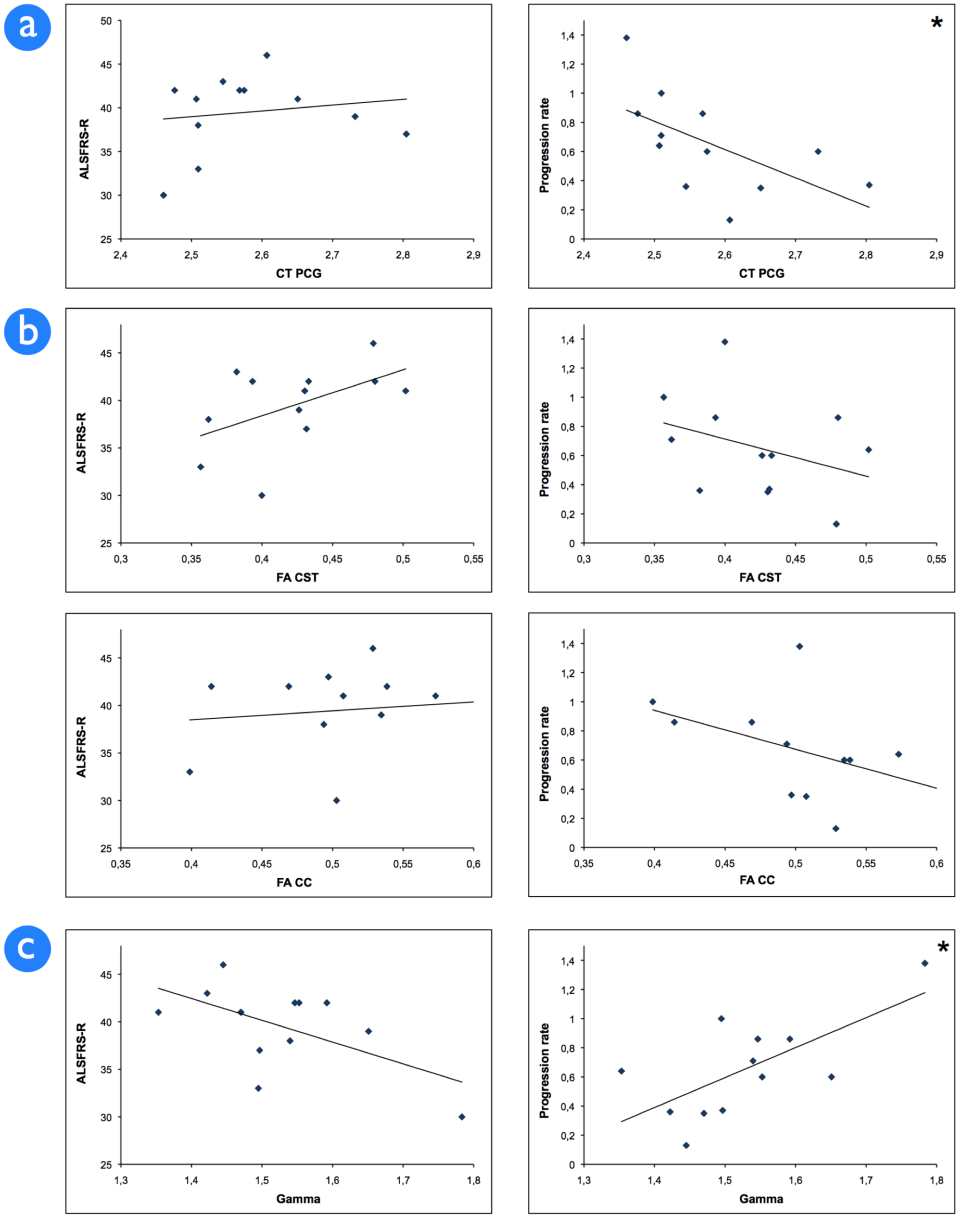
Correlation of imaging parameters with clinical measures. Correlation of ALSFRS-R score and progression rate with; a) cortical thickness in the precentral gyrus (CT PCG) of the clinically most affected side. Cortical thickness is corrected for average whole brain cortical thickness and age; b) FA in the -rostral part of the- corticospinal tract (CST) and FA in the corpus callosum (CC); c) Gamma - or local connectedness - corrected for the number of functional connections and age (threshold 0.30). * p<0.05, ALSFRS-R  =  ALS Functional Rating Scale Revisited. FA  =  fractional anisotropy. CST  =  corticospinal tract. CC  =  corpus callosum.

Functionally, a reduced number of functional connections between the right and left motor cortex was found in ALS. This reduction was not statistically significant (p = 0.14; threshold 0.4), indicating relative sparing of functional connectivity (Figure 3c). Indeed, the overall normalised clustering coefficient gamma, as a measure of local connectedness and local efficiency of the network was unchanged, indicating comparable local functional motor network organisation in patients in comparison to healthy controls. However, focussing on the patient group, gamma values did significantly correlate with disease progression rate in patients with ALS, i.e. stronger interconnected motor networks show a more progressive disease course (Figure 4c).

No association between the structural and functional properties of the motor network was found, by correlating the microstructural integrity of the corpus callosum fibres with the number of functional connections (Supplemental Figure S1). Figure 4 shows the correlation of the motor network measures with clinical markers. Both the average cortical thickness of the precentral gyrus and the level of functional organisation show a significant correlation with progression rate (Figure 4a and 4c respectively).

## Discussion

The present study, based on a network perspective, demonstrates a decline of structural integrity with preserved functional organisation of the motor network in ALS. Firstly, cortical thickness was significantly reduced in the precentral gyrus, known as the primary motor area, in patients with ALS. Secondly, a significant reduction in microstructural organisation was found in the corticospinal tracts (CST) as the major efferent motor conduits to the spinal cord. This involved mainly the rostral part of CST. Furthermore, the corpus callosum, interconnecting the motor network was found to be affected as a whole. Thirdly, exploring the functional connectivity of the motor network in ALS, we found the functional organisation to be intact, however the local connectedness was found to be related with disease progression. This suggests that strong functional connectivity allows for rapid spread of disease.

We observed bilateral cortical thinning of the precentral gyrus in ALS. Interestingly, these effects were most pronounced in two subregions overlapping the somatotopic representation of the hand and leg. These findings are supported by pathological studies which have demonstrated these specific regions to be the areas of the motor cortex with maximal clustering of cortical motor neurons (Betz cells)[24], of which degeneration is considered to be a pathological hallmark of ALS [25]. Indeed, taking the precentral gyrus as a whole, the average cortical thickness in this region was found to be significantly reduced in patients compared to controls. The cortical thickness in the precentral gyrus was shown to be related to disease progression rate, which could suggest increased vulnerability to degenerative effects. Previous studies on gray matter in ALS have shown more widespread atrophy in frontal and temporal regions as well [5], [7], [26], [27]. These studies were exclusively performed using voxel based morphometry, a technique which is known to have an increased signal to noise ratio and is relatively insensitive to cortical atrophy localised in the brain sulci [28]. In other neurodegenerative diseases clinical syndromes were shown to be associated with a specific pattern of cortical thinning which evolves during disease progression marking the clinical relevance of this measure [29]–[31]. As such, cortical thinning patterns could assist in the differentiation between distinct neurodegenerative diseases or identification of subtypes.

With regard to the connectivity of the motor network, it was primarily the rostral part of the CST that was found to be affected. This finding may suggest that the decrease in axonal integrity originates from the cell body, which is in accordance with the observed cortical thinning. These results are supported by the previous observation that cortical hyperexcitability precedes the development of clinical symptoms in pre-symptomatic carriers of a SOD1 mutation [32], thereby suggesting that the early abnormalities in ALS occur within the corticomotorneurons, with anterograde excitotoxicity (often referred to as ‘dying forward’). This aspect of degeneration has been the subject of ongoing debate [32], [33] and longitudinal assessments will need to show whether these effects spread caudally during the course of the disease. In addition, our results showed a severe reduction of white matter integrity in corpus callosum tracts interconnecting the affected left and right cortical regions of the primary motor network. Indeed, callosal dysfunction has been found to be an early phenomenon in ALS [34] and structural involvement of the corpus callosum has been reported in combination with functional impairment [35]. Recently, it has been suggested that the corpus callosum plays an important role in the spread of ALS [33], supporting our functional and structural findings.

We report that patients with stronger connectedness of the motor network show a faster progression of disease, indicating an important role for the functional organisation of the motor network in disease progression. Little is known about how ALS affects the overall functional properties of the motor network. A recent study has suggested reduced connectivity in the default-mode network in ALS, a network linked to high-order cognitive processes and similar to our results no significant overall reduction in inter-hemispheric functional connectivity of the primary motor cortex was reported [16]. As the overall organisation of the motor network was preserved in ALS compared to controls, these results suggest that motor network connectedness may not be implicated in the aetiology of ALS but is involved in the spread of the disease along the structurally and functionally linked primary motor regions [33]. In other neurodegenerative diseases like Alzheimer's disease this hypothesis is supported by the observation that pathological tau-protein spreads in vivo from one brain region to functionally connected spatially distinct regions [36]. In addition, imaging studies have found cortical degeneration in clinical subtypes of dementia occur in distinct functional neural networks [37]. The actual mode of transmission and the applicability of this finding to other neurodegenerative diseases remain, however, subjects for further research. Since, in our study, functional connectivity was found to be of potential prognostic importance it could become a target for therapeutic intervention. Interestingly riluzole, the only drug which delays disease progression is known to increase intracortical inhibition and therefore reduce functional connectivity [38]. An alternative hypothesis for our findings is that the increased local connectedness in patients with a faster disease progression is due to loss of cortical inhibitory interneurons. This effect has been reported previously in studies on excitability of the motor cortex [32], [39]. However, in that case one would expect the average local connectedness to be increased in ALS compared to controls, which is not. Our data did not show a correlation between the functional and structural connectivity in the corpus callosum. This is in acoordance with our opposing results regarding structural and functional connectivity as well as previous studies exploring this correlation [40], [41].

A possible limitation of our study might include the limited sample size and the absence of longitudinal MRI measures. However, ALS is a rare disease with patients often losing ambulation shortly after diagnosis making it difficult to do this type of research in large cohorts of patients. In addition the objective of the present study was to apply multiple imaging techniques to a relatively homogeneous group of patients to explore the central motor network characteristics in relation to clinical markers. Previous studies have too been able to pick up significant functional effects in similar small sample sizes [42]. However, future studies should further clarify the relevance of neural network characteristics regarding vulnerability to neurodegenerative effects. Secondly, using tractography, it may be difficult to differentiate FA effects in the corpus callosum from effects in the rostral part of the CST, as both tracts originate from the same cortical region and cross each other at the level of the corona radiata. This phenomenon of ‘crossing fibres’ is a general technical limitation in the DTI field and the differences found in this region can be interpreted in multiple ways, either the corpus collosum degeneration alone is responsible for these effects, or the CST degeneration or both. Thirdly, little is known about the reproducibility of resting-state fMRI data. However networks with a strong structural connectivity show more reliable functional connectivity across scanning sessions [20].

Our findings reporting on cortical thickness, white matter integrity and functional connectivity, strongly suggest a decline of structural motor network integrity in ALS. Functional connectivity was relatively preserved but was found to be related to disease progression, supporting the hypothesis that neurodegeneration in ALS spreads along functional connections.

## Materials and Methods

### Ethics Statement

All of the subjects gave their informed written consent, in line with the Declaration of Helsinki, and as approved by the local medical ethics committee for research into humans.

### Participants

Twelve patients with ALS (mean age and standard deviation: 48.8±10.6 years) and twelve age and sex-matched healthy controls (age: 49.6±10.5) were included. Subject demographics and relevant clinical information are listed in Table 1. Patients were recruited from the ALS outpatient clinic of the University Medical Centre, Utrecht and were diagnosed with probable lab-supported, probable or definite ALS according to the El Escorial criteria [43]. All patients were treated with riluzole. To minimize confounding by non-ALS-related alterations of the brain we excluded subjects older than 65 years of age and subjects with a history of brain injury, epilepsy, vascular risk factors, psychiatric illness and other systemic diseases. Clinical status of the patients was evaluated using the ALS Functional Rating Scale-Revised (ALSFRS-R) and disease progression rate was assessed (48 - ALSFRS-R-score/disease duration (months)). Additional clinical information is provided in the Supplemental Table S1.

**Table 1:** Study population characteristics.

	ALS	controls
**Sex (M/F)**	10/2	10/2
**Age (years)**	48.8 (33–65)	49.6 (33–64)
**Disease duration (months)**	14.3 (7–30)	
**Time after diagnosis (months)**	5.2 (1–14)	
**Site of onset (spinal/bulbar)**	11/1	
**EE-criteria (probable lab-supported/probable/definite ALS)**	3/8/1	
**ALSFRS-R**	39.5 (30–46)	
**Progression rate**	0.65 (0.13–1.38)	

Abbreviations: M =  male. F =  female. EE  =  El Escorial. ALSFRS-R  =  revised ALS functional rating scale.

### Image acquisition

All imaging data were acquired on a 3 Tesla Philips Achieva Medical Scanner. Imaging included the acquisition of an anatomical T1-weighted image, Diffusion Tensor Imaging (DTI) and resting-state functional MRI (rs-fMRI).

### Cortical thickness: Anatomical T1

A high resolution T1-weighted image was performed for cortical thickness measurements and anatomical reference. Acquisition parameters: 3D FFE using parallel imaging; TR/TE  = 10/4.6 ms, flip-angle 8 degrees, slice orientation: sagittal, 0.75×0.75×0.8 mm voxelsize, FOV  = 160×240×240 mm, reconstruction matrix  = 200×320×320 covering whole brain.

### Structural connectivity: Diffusion Tensor Imaging

In the same scanning session, 2 DTI sets each consisting of 30 diffusion-weighted and 5 diffusion-unweighted scans B = 0 scans (b = 0 s/mm^2^) were acquired to examine structural connectivity of the motor network. Acquisition parameters: DTI-MR using parallel imaging SENSE p-reduction 3; high angular gradient set of 30 different weighted directions [44], [45], TR/TE  = 7035/68 ms, voxel-size 2×2×2 mm, FOV  = 120×120×150 mm, reconstruction matrix  = 120×120×75 covering whole brain, b = 0 s/mm^2^ for the diffusion-unweighted scans and b = 1000 s/mm^2^ for the diffusion-weighted scans, second set with reversed k-space read-out [22], [46].

### Functional connectivity: resting-state functional Magnetic Resonance Imaging

To examine the functional connections of the motor network, resting-state BOLD signals were recorded for a period of 8 min. Acquisition parameters: 3D PRESTOSENSE p/s-reduction 2/2, TR/TE  = 22/32 ms using shifted echo, slice orientation: sagittal, flip-angle 10 degrees, dynamic scan time 0.5 sec, voxel-size 4×4×4 mm, FOV  = 128×256×256 mm, reconstruction matrix  = 40×64×64 (covering whole brain). A short volume acquisition was used to allow for proper sampling of information in the frequency domain up to 1 Hz, effectively minimising the contribution of respiratory and cardiac oscillations (0.3 and >0.8 Hz, respectively), into the resting-state lower frequencies of interest (0.01–0.1 Hz) [47].

### Data analysis and statistics

#### Structural morphology of motor network: Cortical thickness

Cortical thickness measures were performed using the validated and freely available Freesurfer software package (http://surfer.nmr.mgh.harvard.edu/). First, for each individual dataset (both ALS and healthy controls), the anatomical T1-weighted scan was placed into standard space and grey and white matter were segmented. Secondly, for each individual dataset, cortical thickness at every small region of the cortical surfaces was determined by computing the distance between the computed white matter and grey matter surface reconstructions [48]. Thirdly, a group average anatomical image and surface rendering were constructed by normalising all anatomical images to standard space using spherical normalisation. All individual datasets (both of patients with ALS and healthy controls) were normalised to the computed group average anatomical surface, allowing for group comparison between patients with ALS and group matched healthy controls at each small sliver of cortical surface (vertex), covering whole brain.

Whole brain cortical thinning between ALS patients and healthy participants was assessed using General Linear Model (GLM) by testing differences at all vertices. Furthermore, to examine specifically the hypothesised selective thinning of the precentral gyrus in ALS information of the automatic parcellation of the cortical surface [49], [50] was used, parcellating each hemispheric cortical surface in 34 different regions, enabling the computation of an average cortical thickness of each of these region, seperately for the two hemispheres. These 68 regions (each hemisphere covering 34 regions) included the left and right precentral gyrus forming the key regions of the motor network. These primary motor regions were selected for further analysis. Group comparison of the cortical thickness in the precentral gyrus was analyzed using a General Linear Model (GLM) included in the used SPSS software package (http://www.spss.com/software/statistics/). Age and whole brain average cortical thickness were included as covariates to adjust for residual confounding despite global matching [51], [52]. For singly testing the primary motor regions a statistical threshold of α≤0.05 was considered statistically significant. To examine the role of cortical thinning as a possible marker of disease, the level of cortical thickness of the clinically most affected precentral gyrus was correlated with clinical parameters using linear regression.

### Structural connectivity of motor network: Diffusion Tensor Imaging

**Figure 5:**
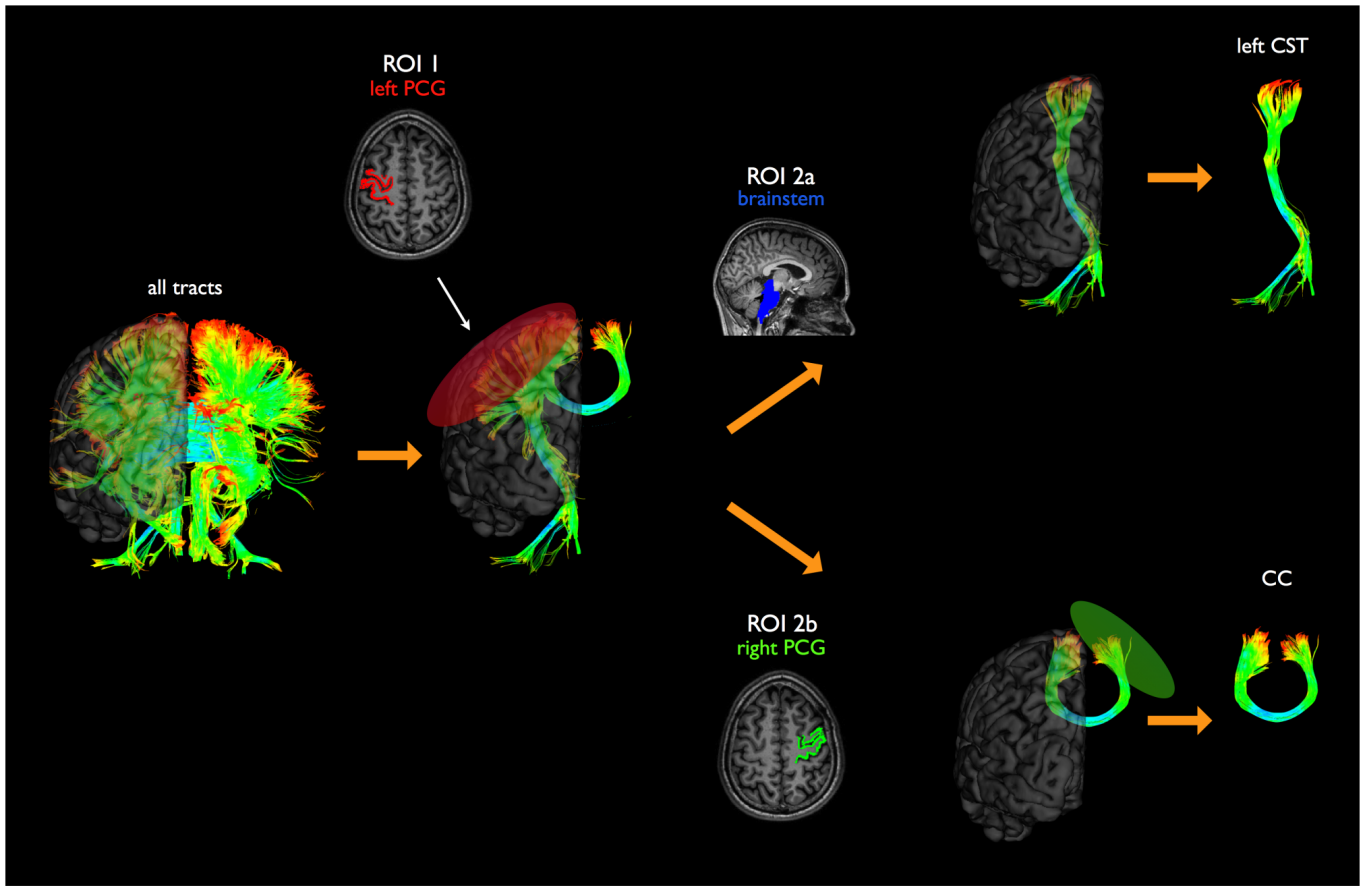
Selection of corticospinal tract (CST) and corpus callosum (CC) fibers. First, all fibres were tracked. Secondly, the left precentral gyrus was selected as region of interest (ROI 1) and the fibres touching this ROI were selected. Thirdly, the brainstem was selected as second ROI (ROI 2a). The left CST was defined by all fibres touching both ROI 1 and ROI 2a. Finally, a third ROI (ROI 2b) was defined as the right precentral gyrus. The CC was defined by all fibres touching both ROI 1 and ROI 2b. The tracts of the right CST were defined in a similar manner. ROI = region of interest. PCG = precentral gyrus. CST = corticospinal tract. CC = corpus callosum.

After post-processing of the DTI white matter tracts of the brain were constructed using the Fibre Assignment by Continuous Tracking (FACT) algorithm [53]. The left and right CST and corpus callosum tract were selected by sorting out those white matter tracts that touched the regions of the primary motor network (Figure 5) [21], [22], [54]. For the tracts of interest, group tracts were constructed to allow group comparison [54] and fractional anisotropy (FA) along the tracts were computed as a measure of white matter integrity of the primary motor tracts [17], [55] (see Supplemental Materials S1). Hypothesized decrease of white matter integrity of the left and right CST and corpus callosum in ALS, were examined by comparison of the individual normalized tracts between patients and the matching healthy controls using multiple linear regression analysis. As a control condition the FA was measured along the visual tracts and the whole brain average FA was computed and compared between patients and controls.

### Functional connectivity of the motor network: resting-state fMRI

To examine possible alterations in the functional communication in the motor network in ALS, resting-state fMRI was acquired [18], [56]. Functional connectivity is defined as the temporal coherence between neuronal signals of anatomically separated brain regions [57], [58]. Resting-state fMRI can be used to map functional connectivity by measuring interregional correlations in spontaneous neuronal synchronization reflected by coherency in low-frequency (<0.1 Hz) blood oxygen level-dependent (BOLD) signal fluctuations [18], . Especially regions of the primary motor network are known to show a high level of synchronisation between their spontaneous resting-state time-series, suggesting ongoing functional communication between motor regions [63]–[65].

To examine the quality of the functional motor network, graph analysis was used, assessing the integrity of functional communication efficiency in the motor network [66], [67]. Representing a dynamic system as a network of regions and their interactions as connections allows for the examination of specific properties of the network. These properties include the level of connectedness or cliqueness of the nodes in the network, indicating how close nodes are connected to their direct neighbours [66]–[68]. This analysis included preprocessing of the resting-state fMRI data and the formation and examination of the organisation of the functional connections between the regions of the motor network (for more details, see Supplemental Materials S1).

In summary, functional connectivity analysis included examining the number of connections providing information on the total level of connectedness of the network, together with the normalised clustering-coefficient gamma providing information about the level of local connectedness and level of local information processing, given by the ratio between the number of connections with the direct neighbours of a node and the total number of possible connections between these neighbours.

#### Statistics

Organisational functional connectivity characteristics (the number of connections and level of local connectedness gamma) were compared between ALS patients and healthy controls using GLM statistics, controlling for possible effects of age.

### Integration of structural, functional and clinical data

The relation between structural and functional measures was explored. The average FA value of the corpus callosum was compared with the number of functional interhemispheric connections using linear correlation. Finally, the main structural and functional measures were compared with the clinical parameters (ALSFRS-R and progression rate) in a similar manner.

## Supporting Information

Materials S1 (0.09 MB DOC)

Table S1 (0.04 MB DOC)

Figure S1 (0.03 MB DOC)
