# Supplementary material for: Motor Network Degeneration in Amyotrophic Lateral Sclerosis: A Structural and Functional Connectivity Study
Source: PLoS One. 2010 Oct 27;5(10):e13664. doi: 10.1371/journal.pone.0013664 (PMC2965124; doi:10.1371/journal.pone.0013664)
Supplement: Materials S1 — (0.09 MB DOC) [file pone.0013664.s001.doc]

**Supplemental Materials S1**

**Motor Network Degeneration in Amyotrophic Lateral Sclerosis: a Structural and Functional Connectivity Study**

Esther Verstraete *^1^, MD; Martijn P. van den Heuvel *^2^, PhD; Jan H. Veldink ^1^, MD PhD; Niels Blanken ^3^, René C. Mandl ^2^, PhD; Hilleke E. Hulshoff Pol ^2^, PhD; Leonard H. van den Berg ^1^, MD PhD

* authors contributed equally

1 Department of Neurology, Rudolf Magnus Institute of Neuroscience, University Medical Centre Utrecht, The Netherlands

2 Department of Psychiatry, Rudolf Magnus Institute of Neuroscience, University Medical Centre Utrecht, The Netherlands

3 Department of Radiology, University Medical Centre Utrecht, The Netherlands

Corresponding author: Leonard H. van den Berg, MD PhD

Address: University Medical Centre Utrecht, Dept of Neurology (HP G03.228), PO Box 85500, 3508 GA Utrecht, The Netherlands. Tel +31887557978, Fax +31302542100, Email: L.H.vandenBerg@umcutrecht.nl

**Materials and Methods**

***Structural connectivity of motor network: Diffusion Tensor Imaging***

*Preprocessing DTI.*

Diffusion Tensor Imaging (DTI) data was pre-processed with the diffusion toolbox of Andersson et al. [1,2] and in-house developed software [3,4]. First of all, of each of the acquired 2 DTI sets, both consisting of 5 diffusion-unwweighted scans (b = 0 s/mm^2^) and 30 diffusion-weighted images (b = 1000 s/mm^2^), were averaged, resulting in 2 average diffusion-unweighted images, one for each set. Secondly, possible susceptibility distortions which are often reported in single-shot EPI images [2], were corrected by combining the information of the two DTI datasets. Possible distortions were estimated by computing a field distortion map based on the two averaged diffusion-unweighted B=0 images, acquired with an opposite k-space read-out direction [2]. The resulting field map was applied to the two sets of 30 diffusion-weighted images, resulting in a single corrected set of 30 diffusion-weighted directions [2]. Finally, correction for eddy-currents and small head movements (linear distortions) was performed [1].

*Reconstruction of white matter tracts.*

To select the CST and corpus callosum tracts of interest fibre tracking was applied. A tensor was fitted to the diffusion profile of each voxel using a robust tensor fit method based on M-estimators [5] and the principal eigenvector of the voxel-specific fitted tensor was selected as the main diffusion direction in that voxel [6]. For each voxel-wise tensor, the fractional anisotropy (FA) was computed, indicating the level of preferred diffusion within that white matter voxel. Next, using the principal diffusion direction of each voxel, the Fibre Assignment by Continuous Tracking (FACT) algorithm was used to reconstruct the total collection of white matter tracts of the brain [6], by starting 8 seeds in each voxel and following the principal diffusion direction from voxel to voxel, until either the fibre exceeded the brain, the fibre trajectory made a sudden change (an angle of >45 degrees) or when the fractional anisotropy value reached below 0.2. This procedure resulted in the reconstruction of all white matter tracts in the brain. The resulting collection of fibres was then used as a starting point for the selection of the tracts of interest [4,7,8].

Selection of CST and corpus callosum tracts of interest.

The tracts of interest in this study were the left and right CST and tracts of the body of the corpus callosum interconnecting left and right precentral gyrus. The left (or right) CST was defined as the fibres that touching the left (or right) precentral gyrus and crossing the brainstem. The corpus callosum tracts were defined as the fibres touching both the left and right precentral gyrus. Selection of the tracts of interest was done using a 4-step procedure [8]. First, the total collection of all reconstructed tracts was selected. Second, to select the left CST, the left precentral gyrus was taken as a region of interest (ROI-1) and all fibres that touched this ROI were selected. Third, the brainstem was selected as a second ROI (ROI-2) and all fibres of the collection of tracts resulting from step 2 that touched ROI-2 were selected. Step 1-3 resulted in the tracts that touched the left precentral gyrus and crossed the brainstem, selecting the left CST. Finally, in step 4, the resulting individual fibres were normalized to the group anatomical image by using non-linear normalization parameters of the subject specific T1-weighted image to the standard group anatomical image and a average group fibre was constructed, to allow comparison between individual datasets [7]. Similarly, to select the right CST, ROI-1 was chosen as the right precentral gyrus. In addition, to select the corpus callosum tracts interconnecting left and right precentral gyrus, ROI-1 was selected as the left and ROI-2 was selected as the right precentral gyrus (Figure 2).

White matter integrity.

To determine the level of integrity of whole brain white matter and, in particular, the selected CST and corpus callosum tracts of interest, the fractional anisotropy (FA) of each white matter voxel and tract were computed. FA reflects the preference of the diffusion profile in a specific direction and ranges between 0 and 1, with 0 indicating no preferred direction of diffusion in a voxel and FA values towards 1 indicating an increasing preference of the water molecules to diffuse in a specific direction. Higher levels of FA represent a higher level of microstructural organisation and decreased values of FA in patients are marking a decrease in white matter integrity [9-11]. For each individual reconstructed fibre tract, the FA profile along the tract was determined by flagging the fibre with the FA values of crossing voxels.

Group tract.

To allow for a point-by-point comparison of the tracts between the group of ALS patients and healthy controls, for each of the tracts of interest (left and right CST and corpus callosum), an average group fibre was constructed by averaging the individual tracts of interest [7]. Each individual tract was then normalized to the average group tract, to allow for point-by-point comparison of the fiber tract integrity values across the group of patients and healthy controls.

***Functional connectivity of the motor network: resting-state fMRI***

Pre-processing resting-state fMRI.

fMRI pre-processing was performed with the SPM5 software package ([http://www.fil.ion.ucl.ac.uk](http://www.fil.ion.ucl.ac.uk/)). First, functional time-series were realigned to correct for possible small head movements. Next, functional time-series were co-registered with the T1-weighted image, for anatomical reference and ensuring overlap with the cortical parcellation maps, and thus allowing the selection of the fMRI voxels overlapping the left and right precentral gyrus. Next, the individual datasets were normalised to allow for spatial group comparison. Resting-state time-series were bandpass filtered to select the resting-state frequencies of interest (0.01-0.08Hz).

Functional network analysis.

To assess the level of functional communication efficiency of the motor network, graph analysis was applied to the functional connectivity data. A graph $G=(V,E)$ is a mathematical description of a network, consisting of nodes V reflecting static points with connections E interconnecting the nodes of the network, illustrated in Figure 1c. From each individual resting-state dataset an individual functional motor network was defined consisting of all primary motor network voxels [12,13]. The steps of this analysis can be described as follows. First, voxels overlapping the left and right precentral gyrus (as defined by the cortical parcellation maps) were selected as nodes. This resulted in a fine grained representation of the motor network in approximately 500 nodes (mean 505; SD 49) for each individual dataset. Next, of all possible voxel-pairs within the motor network the level of functional connectivity was computed as a zero-lag correlation between the voxel-wise resting-state fMRI time-series. This resulted in a N x N connectivity matrix M, with N being the number of motor network nodes, with M(i,j) reflecting the level of functional connectivity between voxel i and voxel j of the motor network with i and j in N. Matrix M was thresholded with a threshold T, setting all connections below this threshold to 0 and all supra-threshold connections to 1. We calculated the network characteristics at ranging thresholds from T=0.3 to T=0.5 in steps of 0.05. This resulted in a binary connectivity matrix B, directly reflecting a connectivity graph $G^{\text{motor}}$, with nodes overlapping all regions of the motor network (i.e. voxels) and connections between voxels functionally connected regions of the motor network.

The level of connectivity in the motor network was examined by computing the total number of connections (k). k reflects the average number of connections of a node in the motor network, indicating on average how strong a node is connected to the other nodes of the network. To correct for differences in the number of nodes, k was normalized by the total number of possible connections that could occur in the network, resulting in an index between 0 and 1.

To examine the level of local clustering of the network as a measure of how strongly interconnected the individual motor network is, the clustering-coefficient $C$ of each of the resulting networks was computed [14-17].The clustering coefficient $C$ indicates the level of how close the nodes of the network are interconnected. It is represented by the ratio between the number of connections between the direct neighbours of a node and the total number of possible connections between these neighbours, reflecting the level of local connectedness of the network. The clustering-coefficient $C_{i}$ of a single node *i* is defined as [17]:

(1)

with $G_{i}^{\text{motor}}$ the local sub-network of connected voxels of node *i*. The overall clustering-coefficient $C$ was defined as the average over all nodes:

(2)

$C$ expresses the level of local connectedness of the network, reflecting how on average the direct neighbours of a node are interconnected themselves.

Typically $C$ is compared to the clustering-coefficient ($C^{\text{random}}$) of a random organised network ($G^{\text{random}}$) with an identical number of nodes and connections as the motor network $G^{\text{motor}}$. The connections between the nodes do not, however, follow a specific organisation but are randomly distributed. The ratio between $C$ and $C^{\text{random}}$ results in the normalised clustering-coefficient gamma. Gamma is typically >1 for networks that are more ordered than random networks, reflecting the level of local efficient information processing within a network [18].For each of the evaluated motor networks (ie for all individual dataset) a collection of h=10 random graphs was computed. $G^{\text{random}}$ was computed as the average over all $C^{\text{random}}(h)$. To ensure that each $G^{\text{random}}(h)$ had the same number of nodes, connections and degree distribution as the original motor network, each $G^{\text{random}}(h)$ was computed by randomizing the connections of $G$ by randomly swapping the connections in $G$ until all connections were distributed keeping the connectivity distribution and similarity of the adjacency matrix intact [19]. Graph randomization and the computation of $C$ and gamma was performed with the Brain Connectivity Toolbox [17]. ([http://www.brain-connectivity-toolbox.net](http://www.brain-connectivity-toolbox.net/)). To correct for a possible effect of variation in the number of k on network organization, the interaction effect of k on gamma was regressed out of gamma using GLM. Finally, to examine the correlation with clinical parameters (within the ALS patients group), k and gamma were corrected for age.

**References**

1. Andersson JL, Skare S (2002) A model-based method for retrospective correction of geometric distortions in diffusion-weighted EPI. NeuroImage 16: 177-199

2. Andersson JL, Skare S, Ashburner J (2003) How to correct susceptibility distortions in spin-echo echo-planar images: application to diffusion tensor imaging. NeuroImage 20: 870-888

3. Mandl RC, Schnack HG, Zwiers MP, van der Schaaf A, Kahn RS, et al. (2008) Functional diffusion tensor imaging: measuring task-related fractional anisotropy changes in the human brain along white matter tracts. PLoS ONE 3: e3631

4. Van den Heuvel MP, Mandl RC, Luigjes J, Hulshoff Pol HE (2008) Microstructural organization of the cingulum tract and the level of default mode functional connectivity. J Neurosci 28: 10844-10851

5. Chang LC, Jones DK, Pierpaoli C (2005) RESTORE: robust estimation of tensors by outlier rejection. Magn Reson Med 53: 1088-1095

6. Mori S, Kaufmann WE, Davatzikos C, Stieltjes B, Amodei L, et al. (2002) Imaging cortical association tracts in the human brain using diffusion-tensor-based axonal tracking. Magn Reson Med 47: 215-223

7. Mandl RC, Schnack HG, Luigjes J, van den Heuvel MP, Cahn W, et al. (2008) Tract-based Analysis of Magnetization Transfer Ratio and Diffusion Tensor Imaging of the Frontal and Frontotemporal Connections in Schizophrenia. Schizophr Bull 36: 778-787

8. van den Heuvel MP, Mandl RC, Kahn RS, Hulshoff Pol HE (2009) Functionally linked resting-state networks reflect the underlying structural connectivity architecture of the human brain. Hum Brain Mapp 30: 3127-3141

9. Budde MD, Kim JH, Liang HF, Schmidt RE, Russell JH, et al. (2007) Toward accurate diagnosis of white matter pathology using diffusion tensor imaging. Magn Reson Med 57: 688-695

10. Kim JH, Loy DN, Liang HF, Trinkaus K, Schmidt RE, et al. (2007) Noninvasive diffusion tensor imaging of evolving white matter pathology in a mouse model of acute spinal cord injury. Magn Reson Med 58: 253-260

11. Budde MD, Kim JH, Liang HF, Russell JH, Cross AH, et al. (2008) Axonal injury detected by in vivo diffusion tensor imaging correlates with neurological disability in a mouse model of multiple sclerosis. NMR Biomed 21: 589-597

12. van den Heuvel MP, Stam CJ, Kahn RS, Hulshoff Pol HE (2009) Efficiency of functional brain networks and intellectual performance. J Neurosci 29: 7619-7624

13. van den Heuvel MP, Stam CJ, Boersma M, Hulshoff Pol HE (2008) Small-world and scale-free organization of voxel-based resting-state functional connectivity in the human brain. Neuroimage 43: 528-539

14. Stam CJ, Reijneveld JC (2007) Graph theoretical analysis of complex networks in the brain. Nonlinear Biomedical Physics 1: 3

15. Reijneveld JC, Ponten SC, Berendse HW, Stam CJ (2007) The application of graph theoretical analysis to complex networks in the brain. Clin Neurophysiol 118: 2317-2331

16. Bullmore E, Sporns O (2009) Complex brain networks: graph theoretical analysis of structural and functional systems. Nature Reviews 10: 186-198

17. Rubinov M, Sporns O (2009) Complex network measures of brain connectivity: Uses and interpretations. Neuroimage 52: 1059-1069

18. Latora V, Marchiori M (2001) Efficient behavior of small-world networks. Physical Review Letters 87: 198701

19. Sporns O, Zwi JD (2004) The small world of the cerebral cortex. Neuroinformatics 2: 145-162
