# Supplementary material for: Motor Network Degeneration in Amyotrophic Lateral Sclerosis: A Structural and Functional Connectivity Study
Source: PLoS One. 2010 Oct 27;5(10):e13664. doi: 10.1371/journal.pone.0013664 (PMC2965124; doi:10.1371/journal.pone.0013664)
Supplement: Table S1 — (0.04 MB DOC) [file pone.0013664.s002.doc]

Supplemental Table S1. Clinical characteristics of the patients with ALS

| **age (years)** | **male/ female** | **site of onset** | **side of onset** | **time to diagnosis (months)** | **disease duration (months)** | **ALSFRS-R** | **disease progression rate** | **diagnosis according the**  **El Escorial criteria** | **predominant UMN/LMN** |
| --- | --- | --- | --- | --- | --- | --- | --- | --- | --- |
| 36 | male | cervical | left | 5 | 10 | 42 | 0.60 | Probable lab supported | LMN |
| 40 | male | cervical | left | 12 | 15 | 46 | 0.13 | Probable lab supported | LMN |
| 53 | male | cervical | right | 6 | 7 | 42 | 0.86 | Probable | LMN |
| 47 | female | bulbar | left | 9 | 15 | 33 | 1.00 | Probable | UMN |
| 62 | male | cervical | right | 12 | 15 | 38 | 0.67 | Definite | UMN |
| 33 | male | cervical | left | 6 | 20 | 41 | 0.35 | Probable | LMN |
| 46 | male | lumbosacral | left | 27 | 30 | 37 | 0.37 | Probable | LMN |
| 44 | male | cervical | right | 6 | 7 | 42 | 0.86 | Probable | UMN |
| 52 | male | lumbosacral | left | 7 | 11 | 41 | 0.63 | Probable lab supported | LMN |
| 44 | female | cervical | left | 11 | 13 | 30 | 1.38 | Probable | LMN |
| 65 | male | lumbosacral | right | 3 | 15 | 39 | 0.60 | Probable | LMN |
| 64 | male | cervical | left | 6 | 14 | 43 | 0.36 | Probable | LMN |

ALSFRS-R: revised ALS funcional rating scale; Probable lab supported: Probable laboratory supported; UMN: upper motor neuron; LMN: lower motor neuron
