# Supplementary material for: Motor Network Degeneration in Amyotrophic Lateral Sclerosis: A Structural and Functional Connectivity Study
Source: PLoS One. 2010 Oct 27;5(10):e13664. doi: 10.1371/journal.pone.0013664 (PMC2965124; doi:10.1371/journal.pone.0013664)
Supplement: Figure S1 — (0.03 MB DOC) [file pone.0013664.s003.doc]

**Supplemental Figure S1: Correlation between structural and functional connectivity in the corpus callosum**

The number of functional interhemispherical connections (threshold 0.4) was plotted against the average FA in the middle portion of the corpus callosum. No significant correlation was observed.
